# Editorial: The immune microenvironment landscape in cerebrovascular diseases

**DOI:** 10.3389/fimmu.2024.1446311

**Published:** 2024-06-19

**Authors:** Zhiqiang Dong

**Affiliations:** ^1^ College of Biomedicine and Health, College of Life Science and Technology, Huazhong Agricultural University, Wuhan, China; ^2^ Research Center of Neurological Diseases, Taihe Hospital, Hubei University of Medicine, Shiyan, Hubei, China

**Keywords:** immune microenvironment, cerebrovascular disease, vascular inflammation, neuro-inflammation, immunomodulation, brain tumor

The immune microenvironment in the brain is shielded by the blood-brain barrier (BBB) and consists of unique cell components, creating a relatively immune-privileged setting (1). Growing evidence has demonstrated that the immune microenvironment plays an important role in the onset, progression, and prognosis of cerebrovascular diseases. Oxidative stress can also impact the immune microenvironment during conditions like ischemic stroke [Zhu et al.; (2)]. Targeting the immune microenvironment has emerged as an effective strategy for the treatment of these diseases. However, the subpopulations and molecular components of the dynamic immune microenvironment demand further characterization. Therefore, the aim of this Research Topic is to gather comprehensive studies related to the immune microenvironment landscape in cerebrovascular diseases.

In this Topic, we have selected six outstanding works from a bunch of articles. Among them, four of these studies delved into the pathophysiology of neuro-inflammation response in stroke, and the other two assessed the communication between neural components and the vascular system.

According to Bourhy et al., brain-immune communication primarily involves the autonomic nervous system, which has the ability to detect and regulate peripheral inflammation in conjunction with the neuroendocrine and limbic systems. Brain-immune crosstalk is a key player in the progression of sepsis and stroke, affecting neurotoxic and neuroprotective mechanisms during both the acute phase and recovery period. Glial cells, as integral components of the neurovascular unit (NVU), are central players in the pathophysiology of neuro-inflammation. Mora et al. demonstrated that NOTCH1 serves as a recognized marker of astrogliosis in neuro-inflammation, highlighting the significance of investigating NOTCH signaling in reactive astrocytes and neighboring NVU cells. This exploration is essential in understanding the pathophysiology of neuro-inflammation conditions and potentially identifying therapeutic targets within this pathway (3).

Microglial cells play a crucial role in interacting with astrocytes and regulating neurotransmitter levels in the brain. Pallarés-Moratalla et al. discussed the responsibilities of microglial cells under physiological conditions and their impact on cerebrovascular diseases. After stroke injury, microglia undergo rapid activation and migration towards the site of injury to aid in brain recovery. Depending on the activation signals, microglia can exert dual roles, either promoting injury or facilitating repair. In the acute phase of ischemic stroke, microglia/macrophages release anti-inflammatory cytokines such as IL-10, protecting neurons against oxygen and glucose deprivation while supporting tissue repair and regeneration. However, after the acute phase, these cells contribute to increased inflammation and cell death by producing cytokines such as IL-6 and TNF-α.

Zhu et al. reviewed the role of glial cells on the occurrence and development of oxidative stress after stroke. Following the stroke, astrocytes and microglia undergo rapid activation, leading to the production of significant levels of reactive oxygen species (ROS) through mitochondrial and NADPH oxidase pathways. On one hand, glial cells can act as a major source of ROS, causing oxidative damage and mediating secondary damage, such as neuroinflammation, excitotoxicity, and blood-brain barrier disruption. On the other hand, ROS influence the behavior of glial cells by activating astrocytes and inducing microglial polarization.

The gut microbiota contributes to host physiology and brain health by generating a variety of metabolites through bacterial *de novo* metabolism and by modifying host-derived molecules (4). Wei et al. reported that after stroke, bidirectional communication between the brain and gastrointestinal tract initiates changes in the gastrointestinal microenvironment. Gastrointestinal immune responses facilitate the migration of gastrointestinal immune cells and cytokines across the damaged BBB, leading to their infiltration into the ischemic brain. Stroke-induced disruptions in gastrointestinal barrier function allow harmful substances to breach the gut mucosal barrier, enter the bloodstream, and eventually reach the brain through the damaged BBB, thereby exacerbating the prognosis of stroke. Therefore, protecting gastrointestinal barrier function following stroke might represent a potential target for improving stroke prognosis.

Neural interactions within the tumor microenvironment play a crucial role in regulating angiogenesis. Shalabi et al. evaluated the molecular interplay and the potential clinical ramifications of manipulating neural elements for the purpose of anti-angiogenic therapeutics within the scope of cancer therapy. They highlighted the presence of neurovascular uncoupling in association with brain tumors. These disruptions interfere with neurovascular coupling, resulting in excitotoxicity caused by an inadequate vascular response, leading to decreased delivery of oxygen and nutrients, resulting in hypoxia and cell death, initiating a chain of events that ultimately triggers angiogenic growth and the development of poorly formed vasculature. This review summarized the potential roles of the signaling pathways and molecules in the development of blood vessels within brain tumors.

Generally speaking, this particular Research Topic successfully hosted reviews which brought additional insights into both understanding and treatments of cerebrovascular diseases. Targeting neuro-inflammation and angiogenesis has already shown promising results in several pre-clinical settings of stroke and glioma. Nevertheless, the efficacy of these approaches is not sufficient yet to achieve a clinically relevant response, thereby necessitating further research in this field to improve patient outcomes.

## Author contributions

ZD: Writing – review & editing, Writing – original draft, Investigation, Conceptualization.
